# Efficient EEG-Based Person Identification: A Unified Framework from Automatic Electrode Selection to Intent Recognition

**DOI:** 10.3390/s26020687

**Published:** 2026-01-20

**Authors:** Yu Pan, Jingjing Dong, Junpeng Zhang

**Affiliations:** 1 School of Electrical Engineering, Sichuan University, Chengdu 610065, China; 2023223035089@stu.scu.edu.cn; 2 Naval Medical Center, Naval Medical University of Chinese PLA, Shanghai 200052, China

**Keywords:** deep learning, automatic electrode selection, person identification, electroencephalogram (EEG), multi-scale bidirectional encoder

## Abstract

Electroencephalography (EEG) has attracted significant attention as an effective modality for interaction between the physical and virtual worlds, with EEG-based person identification serving as a key gateway to such applications. Despite substantial progress in EEG-based person identification, several challenges remain: (1) how to design an end-to-end EEG-based identification pipeline; (2) how to perform automatic electrode selection for each user to reduce redundancy and improve discriminative capacity; (3) how to enhance the backbone network’s feature extraction capability by suppressing irrelevant information and better leveraging informative patterns; and (4) how to leverage higher-level information in EEG signals to achieve intent recognition (i.e., EEG-based task/activity recognition under controlled paradigms) on top of person identification. To address these issues, this article proposes, for the first time, a unified deep learning framework that integrates automatic electrode selection, person identification, and intent recognition. We introduce a novel backbone network, AES-MBE, which integrates automatic electrode selection (AES) and intent recognition. The network combines a channel-attention mechanism with a multi-scale bidirectional encoder (MBE), enabling adaptive capture of fine-grained local features while modeling global temporal dependencies in both forward and backward directions. We validate our approach using the PhysioNet EEG Motor Movement/Imagery Dataset (EEGMMIDB), which contains EEG recordings from 109 subjects performing 4 tasks. Compared with state-of-the-art methods, our framework achieves superior performance. Specifically, our method attains a person identification accuracy of 98.82% using only 4 electrodes and an average intent recognition accuracy of 91.58%. In addition, our approach demonstrates strong stability and robustness as the number of users varies, offering insights for future research and practical applications.

## 1. Introduction

With the rapid development of concepts and technologies such as digital twins, digital currencies, blockchain, and the metaverse, human society is entering an era of digital existence and physical–virtual fusion [1,2]. Along with this shift, the demand for reliable person identification is increasing. As a core enabling technology for interaction between the physical and virtual worlds, brain–computer interfaces (BCIs) have progressed rapidly in recent years, and EEG-based person identification has emerged as an important direction [3,4]. Unlike traditional biometrics such as face, fingerprint, or iris recognition, EEG-based identification leverages the inherent complexity and individuality of neural signals. EEG data also offer unique advantages in security and privacy, as they are extremely difficult to steal or replicate [5,6,7]. Compared with other physiological signals such as electrocardiograms (ECGs) and electromyograms (EMGs), EEG more directly reflects a person’s neural activity and cognitive patterns, while providing high temporal resolution and rich dynamics. Importantly, EEG signals are endogenously generated by the user’s brain activity, giving the user a high degree of control over signal production. These characteristics make EEG-based person identification a promising and secure biometric modality [8].

In recent years, advances in artificial intelligence have accelerated the adoption of deep learning in person identification, attracting considerable interest due to its strong performance and robustness. Compared with traditional pipelines that rely on handcrafted features and classical machine learning algorithms, deep learning methods can learn discriminative representations directly from raw EEG recordings, often improving both computational efficiency and recognition accuracy [9,10,11]. Moreover, EEG is nonlinear and non-stationary, and different cognitive or task-related processes may overlap within a single recording segment. As a result, conventional single-task feature engineering can be insufficient for real-world settings, whereas deep learning-based approaches are better suited to support cross-task person identification.

Although EEG-based person identification has achieved remarkable progress with high classification accuracy [12,13,14], most existing studies overlook two critical issues: the person variability in EEG patterns and the absence of a complete identification and decision-making process. In other words, current EEG-based identification methods often rely on using almost all available electrodes to achieve satisfactory results, neglecting the significant inter-person differences, which leads to unnecessary system complexity and overhead [15]. Moreover, most studies focus only on classifying known persons and fail to consider user intention recognition and dynamic permission management during the identification process [10]. It should be particularly noted that, in this work, intent recognition refers to EEG-based task/activity recognition under controlled experimental paradigms, including motor imagery, motor execution, and eye-state related activities. We employ the term “intent recognition” to emphasize its functional role in our system, where such EEG task-related patterns are interpreted as user intent after identity verification. Therefore, designing an efficient automatic electrode selection mechanism to extract and filter the most relevant features, improve portability, and maintain high performance remains a significant challenge. Additionally, current research often focuses on modeling historical information from EEG data with limited receptive fields or only performs feature extraction in the original spatial domain, ignoring the rich and valuable information contained in the interactions between historical, future, and original spatial features. Another important but underexplored problem is how to infer user intention based on EEG signals. EEG signals carry extensive brain activity information and can be used not only for identity verification but also for task or intention recognition, which holds great significance [11,16].

To address these issues, this article proposes a novel framework that integrates automatic electrode selection, person identification, and intent recognition in EEG-based identification systems. Automatic electrode selection refers to adaptively choosing the most distinctive electrodes based on subject-specific EEG characteristics, minimizing the number of electrodes while maintaining high identification performance. In this work, we present an innovative EEG-based identification framework that supports electrode auto-selection and a dynamic, full-process management approach for person identification and intent recognition. Specifically, we design the AES-MBE network, which integrates automatic electrode selection and intent recognition. This network combines channel attention mechanisms with multi-scale bidirectional feature extraction to capture the most discriminative EEG features and reconstruct different task patterns executed by the same person. We evaluate our proposed framework on the EEG Motor Movement/Imagery Dataset available from PhysioNet [17,18], a large-scale dataset involving 109 subjects performing 4 different tasks. Our experiments demonstrate that the proposed framework achieves state-of-the-art performance in person identification using only 4 electrodes, providing valuable insights and directions for future research. The main contributions of this article are summarized as follows:

1. We propose a novel complete EEG-based person identification and intent recognition framework, capable of automatic electrode selection for each user.
2. We design the AES-MBE network, which extracts effective features from raw EEG signals and performs electrode selection simultaneously.
3. To the best of our knowledge, this is the first attempt to integrate automatic electrode selection and intent recognition in the EEG-based person identification task. Our findings demonstrate that high identification accuracy can be achieved with as few as 4 electrodes, offering a practical solution for the development of portable EEG-based biometric devices.

From a system perspective, the proposed framework is designed as an identity-aware EEG interaction pipeline rather than a collection of independent tasks. In this pipeline, person identification serves as the security and verification layer to confirm the legitimacy of the EEG signal source, while intent recognition is only activated after successful identification. Therefore, intent recognition in our work is not an isolated or parallel task, but a downstream functional module that interprets task/activity-related EEG patterns for verified users. Such a design reflects practical EEG-based biometric and human–computer interaction systems, where identifying who the user is and understanding what the user intends to do are sequential and interdependent processes.

## 2. Related Works

### 2.1. Manually Designed Feature Extraction

Manually designed feature extraction methods are often combined with machine learning approaches. Benefiting from the design and acquisition of EEG features, many machine learning methods, such as support vector machines (SVMs), can achieve satisfactory results. Koike-Akino et al. [19] analyzed event-related potential (ERP) data from 14 EEG channels of 25 subjects. They employed various machine learning techniques and different combinations of dimensionality reduction algorithms to extract EEG features, achieving a maximum accuracy of over 96.7%. Yang et al. [20] applied wavelet transform to extract EEG features for biometric identification, attaining an identification accuracy of 98.24% compared to traditional power spectral features. Chang et al. [21] were the first to propose a novel feature extraction method combining directed functional network analysis with EEG signal complexity from different brain regions. In an experiment where 20 subjects received simultaneous visual and auditory stimuli, they employed SVM classification in the δ-band, achieving a maximum identification accuracy of 90.58%. Jayarathne et al. [22] introduced a novel derived feature, the inter-hemispheric amplitude ratio (IHAR), and utilized ERP data for classification. They tested multiple machine learning algorithms, demonstrating that high accuracy could be achieved even with fewer electrodes. Li et al. [8] extracted effective tensor representations from multi-channel EEG to enhance the discriminability of the feature space. Their proposed method outperformed state-of-the-art approaches when tested on benchmark datasets. Bak et al. [23] focused on person identification using motor imagery (MI) EEG and introduced an EEG-MI approach. They leveraged optimized feature extraction techniques and classifiers to improve person identification accuracy, employing 4 MI-task-related features for machine learning classification. Tatar et al. [24] proposed a nonlinear model-based person identification system. They collected EEG data from 64 channels across 96 subjects and utilized neighborhood component analysis to select the 15 most effective EEG features, achieving high accuracy. Salturk et al. [25] developed a person identification system robust against emotional fluctuations and non-emotional states. Additionally, they examined the impact of different EEG frequency bands and channel regions on biometric identification systems, providing insights for developing cross-task biometric systems.

### 2.2. Deep Learning for Person Identification

With the continuous advancement of deep learning technology, its applications in artificial intelligence have garnered significant attention. Deep learning-based person identification methods eliminate the need for manual feature extraction. EEG signals, which record neural activity, exhibit highly dynamic characteristics and substantial inter-person variability. The application of deep learning enables better abstraction and representation of EEG features, making EEG biometric identification increasingly feasible. Therefore, our research focuses on leveraging deep learning techniques to achieve reliable and efficient EEG-based biometric identification. Zhang et al. [26] employed an attention-based recurrent neural network (RNN) architecture that assigns different attention weights to EEG channels based on their importance, achieving an accuracy of 98.2% on their local dataset (EID-M). In our article, we similarly integrate multiple efficient attention mechanisms. Wang et al. [27] represented EEG signals as graphs based on intra-frequency and cross-frequency functional connectivity estimates. They utilized graph convolutional neural networks (GCNNs) to automatically capture deep intrinsic structural representations from EEG graphs for person identification. Compared to using univariate features directly, the representations extracted from functional connectivity graphs exhibited more robust biometric characteristics. Wilaiprasitporn et al. [28] proposed a cascaded deep learning approach combining convolutional neural networks (CNNs) and RNN, where the CNN captures spatial information from EEG signals, and the RNN extracts temporal features. In our research, we also employ CNN for EEG feature extraction and enhance model expressiveness using a multi-head attention mechanism. Sun et al. [29] introduced a novel method based on one-dimensional convolutional long short-term memory networks (1D-Convolutional LSTM). By leveraging the spatiotemporal characteristics of EEG signals through LSTM, they significantly improved person identification accuracy. When using EEG signals from 16 channels, their proposed network achieved an average accuracy of 99.58%. Building on this foundation, we further introduced automatic channel selection and optimized the model architecture, achieving exceptionally high identification accuracy with only 4 electrodes. Du et al. [30] proposed a transformer-based method for EEG-based person identification, utilizing self-attention mechanisms to extract both temporal and spatial features, demonstrating strong generalization capabilities. Given the powerful feature extraction ability of attention mechanisms, we incorporated a Transformer encoder in our article to enhance model representation capacity. Alsumari et al. [29] introduced a lightweight CNN model with a minimal number of trainable parameters, enabling training and evaluation on limited EEG data. Das et al. [31] proposed an unsupervised feature learning framework based on autoencoders to learn sparse feature representations for EEG-based biometric identification. Their model was tested on a standard public motor imagery EEG dataset comprising 109 subjects, achieving a task-based recognition rate of up to 87.60% and a resting state recognition rate of up to 99.89%. Our article also validated its performance on this dataset and introduced several innovations and improvements. Seyfizadeh et al. [12] employed deep residual neural networks (ResNets) for EEG-based person identification. They utilized continuous wavelet transform to convert one-dimensional EEG signals into two-dimensional spatial images. By integrating frequency and temporal features of EEG signals, their method effectively captured and analyzed intricate details in the data, achieving a classification accuracy of 99.73% on 64 channels from 109 persons. While Seyfizadeh’s research achieved extremely high classification accuracy, it did not consider the impact of electrode count on person identification. In contrast, our work provides significant advantages in this regard. Jin et al. [32] proposed an emotion-EEG-based person identification method with continual learning capabilities, allowing the model to dynamically adapt to evolving person identification requirements. Additionally, they developed a multi-domain coordinated attention transformer as the backbone network, integrating spatial–temporal frequency attention mechanisms with domain coordination mechanisms. Inspired by this, our multi-scale bidirectional encoder facilitates both local and global bidirectional information capture, allowing for adaptive fine-grained feature extraction while establishing cross-temporal representations at a macroscopic level.

### 2.3. EEG Electrode Selection

Most EEG-based person identification studies focus on improving classification accuracy through model and algorithm enhancements while overlooking the impact of electrode count on the convenience of EEG acquisition devices. Due to the complexity and non-linearity of EEG data, some channels may not only fail to contribute useful information for identification but could even introduce noise, adversely affecting system performance. In the field of EEG-based person identification, research on electrode selection has been considered in only a few studies. Therefore, our research emphasizes innovations in this area, providing significant value for future studies. Rodrigues et al. [33] proposed using a binary flower pollination algorithm for EEG channel selection and employed the optimum-path forest classifier to evaluate selection performance. Their method retained 87% accuracy while utilizing fewer than half of the original sensors. Albasri et al. [34] introduced a genetic algorithm to reduce the number of electrodes required for EEG-based identification. Using a public EEG dataset consisting of 109 subjects under open-eye and closed-eye relaxation conditions, they identified approximately 10 out of 64 electrodes as sufficient for accurate identification. Unlike their genetic algorithm-based approach, our research proposes a novel architecture that achieves high identification accuracy using only 4 electrodes. Zhang et al. [35] leveraged a sparse squeeze-and-excitation module to assign weights to EEG channels based on their contributions to MI classification. This led to the development of an automatic channel selection strategy, which, along with time-frequency feature extraction, improved MI classification performance while reducing computational complexity. Similar to Zhang’s work, our research incorporates channel attention mechanisms in a novel framework to quantify electrode importance via weight assignment. Like Albasri et al. [36], Soler et al. [37] applied the NSGA-II optimization algorithm to select the minimal number of electrodes from High-Density EEG data while maintaining source localization accuracy. Their results showed that as few as 6–8 optimized electrodes could match or surpass High-Density EEG localization accuracy in single-source conditions, while 8–16 electrodes were sufficient for multi-source scenarios. Their findings provided valuable insights for our electrode selection research. Tong et al. [38] proposed a channel selection method by integrating an Efficient Channel Attention (ECA) module with a CNN. The ECA module automatically assigned weights to channels based on their importance for BCIs classification, enabling EEG channel importance ranking. Our study builds upon this concept by introducing a multi-scale bidirectional encoder that fully leverages the efficiency of channel attention mechanisms. Wang et al. [39] developed an automatic channel selection method based on spatial filters while quantizing weights and activations to 8-bit precision, with negligible accuracy loss. Their method reduced the number of EEG channels by a factor of 6.4, offering promising insights for future EEG-based identification systems. Amiri et al. [40] proposed a shallow convolutional neural network (SCNN) incorporating temporal and pointwise convolutions to select optimal channels with minimal computational load. After channel selection, a multi-layer fused CNN model classified the EEG signals, achieving a peak accuracy of 81.15%. Our approach builds upon this concept, significantly improving electrode selection and signal classification while incorporating intent recognition, ultimately achieving superior performance across multiple aspects.

## 3. Methodology

### 3.1. System Overview

**Figure 1:**
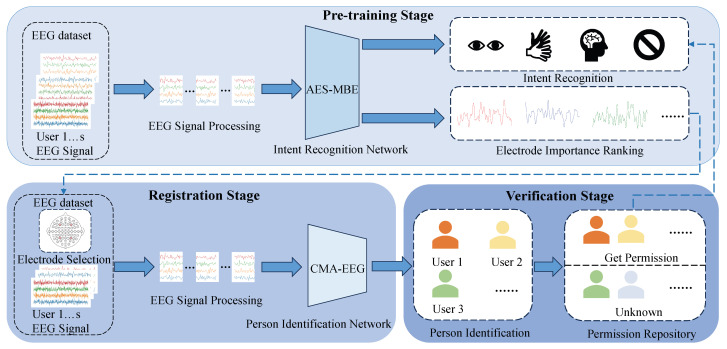
Overview of the proposed framework. The system consists of three stages: the pre-training stage, the registration stage, and the verification stage.

Figure 1 illustrates the proposed framework for EEG-based person identification and intent recognition. The system consists of three stages: the pre-training stage, the registration stage, and the verification stage. In the proposed framework, a subject-specific model is trained separately for each subject that integrates convolutional and channel attention mechanisms [41]. This network is responsible for intent recognition and the automatic selection of electrodes for each user. During the registration stage, the automatically selected electrode signals for each user are used to train a convolutional transformer neural network for person identification [42]. In the verification stage, the classification results obtained are fed into the pre-trained model from the pre-training stage to determine the user’s intent recognition results. As illustrated in Figure 1, the proposed system follows a sequential workflow. Automatic electrode selection and person identification are first performed to obtain identity-related EEG representations. Based on the verified identity, the system subsequently conducts intent recognition, where task/activity-related EEG patterns are interpreted as user intent. This sequential design enables identity-dependent intent analysis rather than generic task recognition. Throughout all stages of the proposed framework, EEG signals are normalized, segmented into 1 s clips, and then fed into the model for training. The rest of this section provides a detailed discussion of the EEG dataset and its preprocessing, the network architecture for intent recognition and automatic electrode selection, and the network architecture for person identification.

### 3.2. Dataset and Processing

The EEG Motor Movement/Imagery Dataset available from PhysioNet [17,18] was employed to validate the proposed method, which consists of data from 109 subjects. Throughout the experiment, all participants were instructed to perform tasks such as resting state, motor imagery, and actual motor tasks. The data were collected using the BCI2000 system (https://www.bci2000.org/, accessed on 1 January 2026), with EEG recorded from up to 64 electrodes following the international 10–10 system at a sampling rate of 160 Hz. For each subject, the dataset contains multiple independent recording runs, where each run corresponds to a continuous EEG acquisition session associated with a specific motor or motor imagery task. These runs are treated as the fundamental units for data partitioning and evaluation in our experiments. The dataset is detailed in Table 1. Before model training and evaluation, each EEG run was segmented into non-overlapping 1 s windows, and each window inherited the corresponding task label of its parent run. All signals underwent preprocessing before use, with time normalization applied to the EEG signals from all 64 channels, as shown in Equation (1). The normalization in Equation (1) is applied per 1 s window and per channel, i.e., the mean and standard deviation are computed within each window. Therefore, no statistics are shared across runs or between training and test sets.

(1) $$I_{i,j}=\frac{Input_{i,j}-\frac{1}{H}\sum_{j=1}^{H}Input_{i,j}}{\sigma_{i}}$$

*i* and *j* denote the indices of the channel and time dimensions of the input EEG data, respectively. *H* denotes the number of samples in a 1 s window (H=160), and $\sigma_{i}$ represents the standard deviation of channel *i*. The normalized signal $I_{i,j}$ will then undergo further processing. Each EEG signal was sliced into segments, with 160 samples per segment, corresponding to a duration of 1 s.

### 3.3. Architecture of AES-MBE Backbone Network

We have observed that most previous studies in the field of EEG-based person identification rely on models trained with electrodes placed at fixed positions. Such manual and subjective electrode selection has significant limitations—improper selection can degrade model performance and increase redundancy and noise. Secondly, most deep learning models adopt a unidirectional approach to data processing, utilizing only current and past information while ignoring future time steps. While this approach is sufficient for many tasks, such as natural language processing (NLP), where models rely on past information for generative autoregressive tasks, it is inadequate for EEG classification tasks. EEG analysis requires processing information from both past and future time steps simultaneously, making unidirectional modeling insufficient. Finally, most existing studies on EEG-based person identification focus on isolated tasks, such as either person identification or intent recognition, and fail to achieve the integration of both.

**Figure 2:**
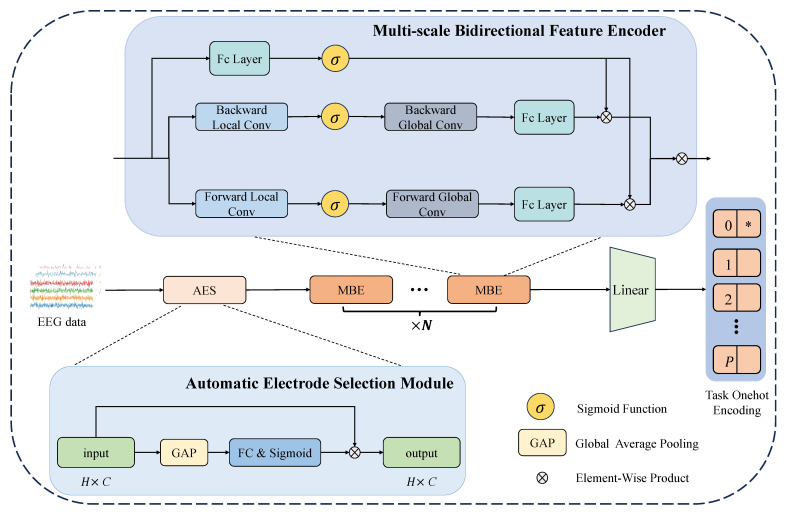
AES-MBE backbone network, including the internal processing pipeline of the automatic electrode selection (AES) module and the multi-scale bidirectional feature encoder (MBE). The asterisk (*) indicates the final classification output.

Inspired by the above observations, we propose the AES-MBE model as our primary network, with its overall architecture illustrated in Figure 2. This network consists of two main modules. The first is the automatic electrode selection module, which leverages a channel attention mechanism to select the most discriminative electrodes specific to each person, thereby enhancing the performance of the subsequent person identification model. The second module is a multi-scale bidirectional feature encoder designed to capture both past and future temporal information in EEG signals. The notion of forward and backward temporal modeling, including bidirectional convolutional architectures, has been explored in prior studies for sequence representation and time-series analysis [43,44]. Our work does not introduce a new convolution operator and instead we build upon these existing bidirectional modeling ideas and adapt them to a unified multi-scale convolutional framework tailored for EEG signal characteristics.

To the best of our knowledge, this is the first attempt to integrate a multi-scale bidirectional convolutional encoder with automatic electrode selection into a unified backbone network for EEG-based person identification and intent recognition. By jointly exploiting global and local temporal dependencies as well as subject-specific electrode importance, the proposed AES-MBE backbone enables accurate person identification while simultaneously supporting intent recognition within a single end-to-end framework.

Finally, all outputs are mapped to one-hot encoded vectors. This task-independent encoding approach facilitates multi-task learning, simplifies the overall network structure, and improves both the robustness and efficiency of the model. Table 2 illustrates the overall architecture of AES-MBE.

#### 3.3.1. Automatic Electrode Selection Module

In person identification tasks, not all EEG channels contribute equally due to inter-subject variability. Therefore, automatic electrode selection aims to identify a compact set of subject-discriminative electrodes while reducing redundancy and computational overhead. To this end, we employ a subject-specific pre-training model in which the AES module performs channel importance estimation. The AES module is built on a channel-attention mechanism that learns inter-channel dependencies and produces adaptive channel-wise weights during training, emphasizing informative electrodes and down-weighting less relevant ones. As a result, electrode selection is achieved in a data-driven manner without explicit dimensionality reduction. It is important to clarify that the automatic electrode selection is performed at a subject level during a dedicated pre-training stage. Specifically, for each subject, the AES module is trained to learn an importance score for all 64 electrodes, resulting in a subject-specific electrode importance ranking. Once this pre-training stage is completed, the learned ranking for each subject is fixed and reused for constructing inputs in the subsequent person identification stage.

The input data are preprocessed, and the EEG signals fed into the AES Module can be regarded as a H×C matrix, where *H* represents the sequence length (i.e., the number of temporal samples) of the EEG data and *C* represents the number of channels in the EEG data, and the AES Module first pools the global mean of the EEG along the temporal dimension to help the model aggregate the global information, thus generating a global response feature map of the channel features. The global response feature map of the channel features can be expressed as Equation (2):

(2) $$z_{c}=\frac{1}{H}\sum_{i=1}^{H}x_{i,c}$$

Here, $x_{i,c}$ denotes the EEG amplitude of the *c*-th channel at the *i*-th temporal sample, where $z_{c}\in R^{C}$ is the global mean feature vector obtained by averaging along the temporal dimension, with each element reflecting the response of the corresponding channel. Subsequently, after a fully connected layer, the attention weights of each channel are computed through the channel attention mechanism, the weight matrix of the fully connected layer is defined as follows:

(3) $$W_{fc}\in R^{C\times C}$$

Here, $W_{fc}$ denotes the learnable weight matrix of the fully connected (FC) layer in the AES module. In our implementation, $W_{fc}$ is randomly initialized at the beginning of training using the default initialization scheme of PyTorch (v2.3.1; https://pytorch.org/; accessed on 1 January 2026) torch.nn.Linear, i.e., Kaiming uniform initialization. Specifically, each element of $W_{fc}$ is sampled from a uniform distribution:

(4) $$W_{fc}∼U\left(-\frac{1}{\sqrt{C}},\;\frac{1}{\sqrt{C}}\right)$$

Here *C* denotes the number of input channels, and U(a,b) denotes the continuous uniform distribution on the interval [a,b]. The weight matrix $W_{fc}$ is subsequently optimized via backpropagation during network training. For clarity, we use $w_{j,c}$ to denote an element of $W_{fc}$, where the column index *c* corresponds to the *c*-th input channel and the row index *j* corresponds to the *j*-th output unit of the FC layer.

Let $z\in R^{C}$ denote the channel-wise descriptor vector obtained by the global average pooling (GAP) operation, where $z_{c}$ is the *c*-th element of *z*.

The channel attention weights are then computed as:

(5) $$\alpha_{c}=\sigma \left({\left(W_{fc}\cdot z\right)}_{c}\right)$$

Here $\alpha_{c}$ represents the attention weight of the *c*-th channel, σ(·) denotes the activation function, and ${\left(\cdot \right)}_{c}$ indicates the *c*-th element of a vector. We use the sigmoid function, which constrains the channel attention weights $\alpha_{c}$ within the range [0,1].

Learning through the fully connected layer ensures that the module takes into account the importance of each channel and computes the importance of each channel feature. The channel weights will be weighted to the input EEG feature maps and will not change the dimensionality of the data as shown in Equation (6), where *X* represents the input EEG feature matrix, $X^{\prime }$ denotes the re-weighted EEG feature matrix, α is the attention weight vector for each channel, and ⊙ indicates element-wise multiplication along the channel dimension.

(6) $$X^{\prime }=\alpha ⊙X$$

#### 3.3.2. Proposed Electrode Selection Method

As previously mentioned, we utilize the AES Module to perform automatic channel selection, with the core mechanism being the channel attention strategy. During model training for different subjects, the channel attention mechanism automatically optimizes the attention weights for each channel based on model performance, assigning higher weights to more informative channels. Through this subject-specific optimization process, the AES module progressively captures the relative relevance of each channel with respect to person identification, thereby inherently accounting for inter-subject variability and enabling personalized electrode preference.

After model convergence, our objective is to derive a subject-specific yet fixed electrode ranking that can be extracted once and reused in subsequent stages. The fixed weights of the fully connected (FC) layer are extracted, and the importance score for each channel is calculated according to Equation (7). Although the channel attention mechanism produces input-dependent gating coefficients during inference, these coefficients may vary across EEG segments and do not directly provide a deterministic channel ranking without further data aggregation. Therefore, to enable stable and reproducible post-training electrode selection, we estimate channel importance using the learned FC weights as a proxy for channel relevance accumulated during training. The channels are then ranked based on these scores, and the top *n* channels are selected as the subject-specific optimal electrodes, where *n* can be predetermined by the researcher. Once selected, this electrode subset remains fixed for the subject and is used throughout the downstream person-identification process. This method ensures that for a fixed number of electrodes, the most discriminative channels, with the greatest differences among persons, are automatically selected, thereby enhancing the person identification performance.

(7) $$s_{c}=\sum_{j=1}^{D}\left|w_{j,c}\right|$$

In Equation (7), $w_{j,c}$ denotes the FC weight connecting the *c*-th input channel to the *j*-th output unit, *D* is the number of output units of the FC layer (in our implementation, D=C), and $s_{c}$ represents the resulting importance score of channel *c*. Therefore, the term $w_{c,c}$ simply corresponds to one element of the learned weight matrix (a diagonal entry when D=C), i.e., the connection weight from the *c*-th input channel to the *c*-th output unit, and it is treated the same as other $w_{j,c}$ terms in the aggregation. The score is computed by aggregating the magnitudes of all FC weights connected to channel *c*. It should be noted that this formulation does not aim to measure the exact expected contribution of a channel for an individual EEG segment; instead, it serves as a stable, data-agnostic proxy of subject-level channel relevance learned during training, which is particularly suitable for fixed electrode ranking after model convergence.

#### 3.3.3. Multi-Scale Bidirectional Feature Encoder

In previous EEG studies, most approaches have only considered historical information. However, given the unique characteristics of EEG signals—which differ significantly from typical one-dimensional time series that rely solely on current and past information—this simplification is inadequate. EEG signals are highly complex, nonlinear, and often exhibit a low signal-to-noise ratio. Thus, an urgent challenge is how to effectively extract more meaningful information from these signals. To address this, we designed a multi-scale bidirectional encoder along the temporal dimension, capable of modeling EEG data in both forward and backward directions. Figure 2 provides an intuitive overview of the complete processing pipeline of the proposed multi-scale bidirectional feature encoder, explicitly illustrating the information flow from multi-scale temporal convolutional branches to bidirectional encoding and subsequent feature fusion. This structure allows the model to learn temporal dependencies more effectively and capture features at different time scales. Specifically, as shown in Figure 2, the input EEG signals are processed by multiple temporal convolution branches with different receptive field sizes; the resulting features are then encoded in both forward and backward directions and fused to form the final temporal representation. It should be noted that the bidirectional convolutional design operates on fixed-length EEG segments and therefore assumes a window-based processing scheme. This setting is commonly adopted in EEG analysis and biometric systems, where signals are segmented into short temporal windows for feature extraction. While the backward branch requires access to future samples within the same window, it does not affect the applicability of the proposed method to offline or block-based processing scenarios considered in this work. In addition, within the context of task fusion, both macro-level tasks and micro-level tasks require feature extraction at different scales, as they present varying temporal dependencies. Therefore, we introduce convolutional kernels with multiple receptive field sizes in the multi-scale bidirectional encoder, enabling the simultaneous capture of short-term local patterns and long-term global dependencies.

Specifically, given the input EEG signals $X\in R^{H\times C}$, where *H* represents the sequence length and *C* represents the number of channels, we first perform multi-scale one-dimensional convolution operations with different kernel sizes $k_{1},k_{2},\ldots ,k_{n}$ to extract features at multiple temporal scales. The feature extraction process is formulated as Equation (8).

(8) $$F_{k_{i}}=\mathrm{Conv}1D_{k_{i}}\left(X\right),\;i=1,2,\ldots ,n$$

Here, $\mathrm{Conv}1Dk_{i}\left(\cdot \right)$ denotes a one-dimensional convolution operation with kernel size $k_{i}$, and $F_{k_{i}}$ represents the feature map extracted at scale $k_{i}$.

Next, we concatenate and fuse the extracted features from different scales to form a comprehensive temporal dependency representation, as shown in Equation (9).

(9) $$F_{\mathrm{multi}}=\mathrm{Concat}\left(F_{k_{1}},F_{k_{2}},\ldots ,F_{k_{n}}\right)$$

To further capture bidirectional dependencies in the time series, we introduce a bidirectional convolution mechanism, extracting features from both forward (left-to-right) and backward (right-to-left) directions. The computation is given by Equation (10).

(10) $$\begin{array}{cc} F_{\mathrm{forward}} & =\mathrm{Conv}1D_{\mathrm{forward}}\left(F_{\mathrm{multi}}\right),\\ F_{\mathrm{backward}} & =\mathrm{Conv}1D_{\mathrm{backward}}\left(F_{\mathrm{multi}}\right) \end{array}$$

In this process, Conv1Dforward(·) captures features in the forward direction, while Conv1Dbackward(·) captures features in the backward direction. Finally, we integrate the forward features, backward features, and the linear projection of the original input through a fully connected layer, enhancing the model’s capability to learn bidirectional temporal dependencies. The fusion is expressed as Equation (11).

(11) $$F_{\mathrm{final}}=\mathrm{Concat}\left(F_{\mathrm{forward}},F_{\mathrm{backward}},W_{fc}\cdot X\right)$$

Here, $W_{fc}$ denotes the weight matrix of the fully connected layer. Through this design, our multi-scale bidirectional encoder is able to adapt to the temporal patterns required by different tasks, effectively capturing both short-term local features and long-term global dependencies, thus enabling more refined EEG feature extraction.

### 3.4. Person Identification Model

**Figure 3:**
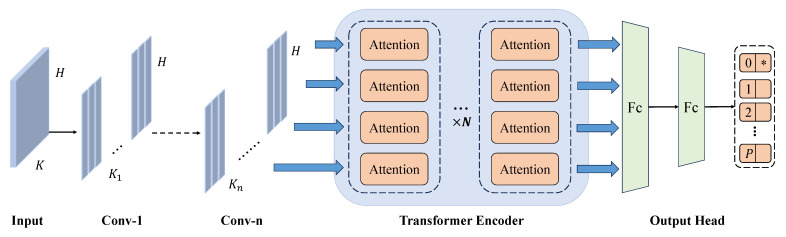
Overall architecture of the proposed CMA-EEG-based person identification model, which consists of multiple CNN convolutional layers and a Transformer encoder composed of multi-head attention mechanisms. The asterisk (*) indicates the final classification output.

In the registration stage, we define a hybrid convolutional and Transformer encoder classification model, which aims to fully combine the advantages of CNN in local spatial feature extraction and the ability of the Transformer encoder in long-range dependency modeling [42,45]. Since the Transformer encoder is fundamentally built upon multi-head self-attention, this hybrid CNN–Transformer-based person identification model is referred to as the Convolutional Multi-Head Attention (CMA) model throughout the paper. To improve EEG-based person identification performance, the architecture is shown in Figure 3.

Given a sample belonging to subject *s*, the input construction proceeds as follows. First, the subject-specific electrode importance ranking learned in the pre-training stage is retrieved. Then, the indices of the top-*K* electrodes (e.g., K=4) are selected from the full set of 64 electrodes. The corresponding raw EEG signals are gathered from the original multi-channel recording and stacked into a *K*-channel input tensor. To ensure a consistent and reproducible channel ordering, the selected electrodes are re-indexed into a canonical order according to their electrode indices (ascending order) before stacking. As a result, the person identification network always receives a fixed-dimensional input of size K×H, where *H* denotes the temporal length of the EEG data. This subject-specific electrode set is determined once during an enrollment/calibration phase and then fixed for subsequent sessions.

Although the physical electrode identities selected for different subjects may vary, this does not introduce a mismatch with the downstream multi-class person identification model. The classifier is a single unified network trained across all subjects, and it only requires a fixed number of input channels rather than fixed physical electrode identities. The subject-specific electrode selection reflects inter-subject variability in discriminative EEG channels, while the input representation fed to the classifier remains consistent in dimensionality.

Specifically, given an input EEG signal: $X\in R^{C\times H}$ where *C* denotes the number of channels and *H* represents the length of EEG data. First, a series of one-dimensional convolutional layers are applied to extract local features, as Equation (12):

(12) $$\begin{array}{c} F_{l}=\mathrm{BN}\left(\mathrm{Conv}1D_{l}\left(F_{l-1}\right)\right),\;l=1,2,\ldots ,n,\\ F_{0}=X\; \end{array}$$

Here, $F_{l}$ denotes the output feature of the *l*-th convolution layer, $\mathrm{Conv}1D_{l}\left(\cdot \right)$ represents the one-dimensional convolution operation, BN(·) is batch normalization. After *n* convolution layers, we obtain Equation (13).

(13) $$F_{n}\in R^{C^{\prime }\times H^{\prime }}$$

$C^{\prime }$ is the number of channels after the final convolution layer, and $H^{\prime }$ is the downsampled time dimension.

The resulting feature map is then fed into a Transformer encoder. We first transpose the feature matrix as in Equation (14).

(14) $$Z_{0}=F_{n}^{H}\in R^{H^{\prime }\times C^{\prime }}$$

In the Transformer encoder, Multi-Head Self-Attention (MHSA) is employed to capture global temporal dependencies. For the input $Z_{l-1}$ of the *l*-th Transformer layer, the query, key, and value matrices are as shown in Equation (15).

(15) $$Q=Z_{l-1}W_{Q},\;K=Z_{l-1}W_{K},\;V=Z_{l-1}W_{V}$$

$W_{Q},W_{K},W_{V}\in R^{C^{\prime }\times d}$ are learnable projection matrices, and *d* is the dimensionality of each attention head. The self-attention mechanism is then computed as Equation (16).

(16) $$\mathrm{Attention}\left(Q,K,V\right)=\mathrm{softmax}\left(\frac{QK^{T}}{\sqrt{d}}\right)V$$

The multi-head attention output is computed by concatenating the results of *h* attention heads as Equation (17).

(17) $$\mathrm{MHSA}\left(Z_{l-1}\right)=\mathrm{Concat}\left(head_{1},head_{2},\ldots ,head_{h}\right)W_{O}$$

Each head is defined as Equation (18).

(18) $$head_{i}=\mathrm{Attention}\left(Q_{i},K_{i},V_{i}\right)$$

$W_{O}$ is the output projection matrix.

The forward pass of each Transformer encoder layer is given by Equation (19).

(19) $$\begin{array}{c} Z_{l}^{\prime }=\mathrm{LN}\left(Z_{l-1}+\mathrm{MHSA}\left(Z_{l-1}\right)\right),\;l=1,2,\ldots ,L\\ Z_{l}=\mathrm{LN}\left(Z_{l}^{\prime }+\mathrm{FFN}\left(Z_{l}^{\prime }\right)\right)\; \end{array}$$

Here, LN(·) denotes layer normalization and FFN(·) is a position-wise feed-forward network. *L* is the total number of Transformer layers.

After passing through the Transformer encoder, the output sequence is processed by global average pooling as Equation (20).

(20) $$F_{\mathrm{out}}=\mathrm{AvgPool}\left(Z_{L}\right)\in R^{C^{\prime }}$$

Finally, the pooled feature vector is fed into a fully connected (FC) layer for classification as Equation (21).

(21) $$y=W_{\mathrm{fc}}\cdot F_{\mathrm{out}}+b_{\mathrm{fc}},\;y\in R^{K}$$

The predicted probability distribution over the *K* classes (corresponding to different persons) is computed using softmax as Equation (22).

(22) $$P_{i}=\frac{e^{y_{i}}}{\sum_{j=1}^{K}e^{y_{j}}},\;i=1,2,\ldots ,K$$

Here $P_{i}$ represents the probability of the input belonging to class *i*. The final output is then mapped to a one-hot encoded vector as Equation (23).

(23) $$O_{i}=\left\{\begin{array}{cc} 1, & \mathrm{if}\;i=\arg\max_{j}P_{j}\\ 0, & \mathrm{otherwise} \end{array}\right.$$

## 4. Experimental Setup

### 4.1. Experimental Protocol

Considering EEG signals as non-stationary time-series data, temporal variations can significantly affect model performance. Therefore, it is necessary to evaluate the proposed framework using EEG data collected at different time intervals. To faithfully simulate real-world EEG-based person identification scenarios while avoiding temporal data leakage, all experiments were conducted using a strict run-wise (*n*-fold) cross-validation protocol rather than window-wise random splitting. Specifically, EEGMMIDB provides multiple independent runs (records) for each subject. In each fold, runs were first split into mutually exclusive training and testing sets with no overlap. Only after the run-wise split was fixed were individual runs segmented into non-overlapping 1 s windows for model training and evaluation. Consequently, all windows originating from the same run appeared exclusively in either the training set or the testing set within a given fold, effectively preventing temporal correlation and data leakage between training and testing samples. To ensure reproducibility and to avoid ambiguity in run partitioning, we adopt a fixed run-wise three-fold split with predefined run indices. Specifically, we use different fixed split templates for the pre-training stage (subject-specific AES/intent learning) and the registration stage (closed-set person identification). In all cases, runs in the training set and the test set are strictly non-overlapping within each fold, and 1 s windows are generated after the run split is fixed.

In order to reflect the robustness and reliability of the experimental results and to minimize the effect of random variables, we repeat all experiments 20 times and ensure the same random seed and training hyperparameter settings. Secondly, we use accuracy rate, precision rate, recall rate, false acceptance rate, false rejection rate, and equal error rate to evaluate the model, which basically includes the mainstream evaluation metrics in the field of person identification.

The specific experimental design is divided into three parts: the pre-training stage, the registration stage, and the verification stage, as shown in Figure 1. All person identification experiments in this work are conducted under a closed-set setting, where the identities appearing in the test set are included in the set of subjects seen during training. Accordingly, the subject-specific electrode importance rankings are learned from the training data and directly applied to the test samples of the same subject. Handling previously unseen (unknown) subjects would require an additional enrollment or calibration stage to estimate electrode importance.

In this work, the proposed model is trained as a closed-set multi-class user identification network with a softmax output over K=109 subjects, using one-hot encoded identity labels. In addition to identification accuracy, we further evaluate the model under a derived identity verification protocol based on the same multi-class outputs, which enables the computation of verification metrics such as FAR, FRR, and EER.

Specifically, for each test sample $x_{i}$ with ground-truth identity $y_{i}\in \left\{1,\ldots ,K\right\}$, we form verification trials by pairing $x_{i}$ with a claimed identity c∈{1,…,K}. To obtain a deterministic and reproducible genuine/impostor pairing strategy, we enumerate all possible claims for each $x_{i}$, resulting in exactly one genuine trial $\left(x_{i},c=y_{i}\right)$ and K−1 impostor trials $\left(x_{i},c\neq y_{i}\right)$. The verification score for a trial $\left(x_{i},c\right)$ is defined as the softmax posterior corresponding to the claimed identity:

(24) $$s\left(x_{i},c\right)=p_{\theta }\left(c|x_{i}\right).$$

A binary accept/reject decision is made by thresholding this score with τ∈[0,1]: the claim is accepted if $s(x_{i},c)\geq \tau$, and rejected otherwise. Rather than being fixed or tuned during training, τ is swept over its valid range during evaluation (e.g., over all unique score values) to characterize verification behavior at different operating points.

Let $G=\{\left(x_{i},c\right):c=y_{i}\}$ denote the set of genuine trials and $I=\{\left(x_{i},c\right):c\neq y_{i}\}$ the set of impostor trials. The false acceptance rate (FAR) and false rejection rate (FRR) at threshold τ are computed as:

(25) $$\mathrm{FAR}\left(\tau \right)=\frac{\left|\{\left(x_{i},c\right)\in I:s\left(x_{i},c\right)\geq \tau \}\right|}{\left|I\right|},\;\mathrm{FRR}\left(\tau \right)=\frac{\left|\{\left(x_{i},c\right)\in G:s\left(x_{i},c\right)<\tau \}\right|}{\left|G\right|}.$$

The equal error rate (EER) is defined as the operating point where FAR(τ)=FRR(τ). In practice, we compute EER using linear interpolation between the two thresholds surrounding the intersection, or equivalently by selecting the threshold that minimizes |FAR(τ)−FRR(τ)|. This protocol consistently evaluates verification performance using the outputs of the multi-class identification model, without introducing an additional verification-specific classifier, and remains within the same closed-set of enrolled subjects.

#### 4.1.1. Pre-Training Stage

In the pre-training stage, the AES module is trained separately for each target subject. Here, the term “current” refers exclusively to the target subject of the ongoing pre-training round, rather than a time-varying state, while all remaining subjects are treated as “other subjects”. In this phase, the model is trained for each of the 109 persons in the dataset and evaluated independently. The primary objective of this stage is not to establish a standard intent-recognition benchmark, but to learn subject-discriminative channel attention patterns that will be used for subsequent subject-specific electrode selection. Accordingly, the pre-training task is formulated to explicitly strengthen the separability between the target subject and other subjects: EEG samples from the target subject are labeled by motor-related states (motor execution, motor imagery, and resting), while EEG samples from all other subjects are grouped as non-target (other-subject) data. Introducing data from other subjects provides negative reference samples that enhance inter-subject discrimination for electrode selection, rather than defining an additional motor-intent category.

In this stage of the experiment, three-fold cross-validation is used to ensure the robustness of the model. Specifically, EEGMMIDB provides 14 complete EEG runs per subject, and cross-validation is conducted at the run level using a fixed three-fold split template. In each fold, 5 runs are held out for testing and the remaining runs are used for training. Importantly, runs 1–2 correspond to resting state recordings in EEGMMIDB; therefore, a run index of 1 or 2 may appear in the test set of certain folds, which is intentional and ensures that resting state data are also evaluated under strict run-wise hold-out.

All the data are processed as described in the previous section. After the run-wise split was fixed, each EEG run was segmented into non-overlapping 1 s windows, and each segment inherited the corresponding category label of its parent run. As a result, all EEG segments originating from the same run appeared exclusively in either the training set or the testing set within a given fold, which effectively prevents temporal correlation and information leakage between the two sets. After convergence, the learned attention-related parameters are then used to derive a fixed, subject-specific electrode ranking in the proposed AES module.

#### 4.1.2. Registration Stage

In the registration stage, a closed-set person identification model is trained using all 109 subjects as 109 identity classes, while adopting the fixed, subject-specific electrode configuration obtained from the pre-training stage. The EEG data of 109 subjects are used as 109 categories in the person identification task at this stage. Following the same run-wise evaluation principle, the registration stage adopts a fixed three-fold run-level split template. In each fold, 4 runs are held out for testing and the remaining 10 runs are used for training, with strictly non-overlapping runs between training and testing. Runs used for training and testing are category-balanced at the run level to mitigate overfitting and ensure fair evaluation across different task-related runs.

For each training iteration of the network in the experiment, the training dataset is normalized, shuffled, and batched. Each batch contains 256 sets of $160\times N_{\mathrm{chan}}$ EEG samples, where the number of channels can be specified by the user or automatically selected by the electrodes. The training can be stopped early either after 1000 epochs or when the loss function no longer decreases. Input EEG segments are normalized per 1 s window and per channel using Equation (1) before being fed into the network.

Note that the exact run-level hold-out templates differ between the pre-training and registration stages because the two stages serve different learning objectives and thus adopt different fixed partition templates. Nevertheless, both stages consistently enforce non-overlapping run-wise splits, and all windowing is performed only after the run split is fixed, ensuring a leakage-free and reproducible evaluation protocol.

#### 4.1.3. Verification Stage

We determine whether the two EEG features belong to the same person by the position of the 109-dimensional one-hot encoding output by the model, and when we obtain the classification result output by the model, we will send the result of this determination to the permission repository for further judgment, so as to obtain the permission of the person, and at the same time, we input the model obtained by the person in the pre-training stage to obtain the result of its intent recognition.

### 4.2. Implementation Details

Python 3.8 was used for implementation, and PyTorch [46] was used as the deep learning framework, unless otherwise stated. All experiments were conducted on a workstation equipped with an NVIDIA GeForce RTX 3070 Ti GPU, an Intel Core i9-11900K CPU (@3.50 GHz), and Windows 11. Each experiment starts with a learning rate of 0.0001, using SGD with momentum as the optimizer, and a batch size of 256, which can be set dynamically depending on the GPU memory size.

## 5. Results and Discussion

Considering that our proposed method innovatively integrates EEG-based person identification with intent recognition, and introduces, for the first time, an automatic electrode selection method for use within our system, we evaluate the performance of our system from two perspectives: person identification and intent recognition. In order to demonstrate the superiority of our proposed backbone network compared to existing studies, we conduct comparisons with multiple baseline models to highlight the high performance of our approach. Furthermore, we examine the impact of the proposed automatic electrode selection method on the overall system performance. Visualization of results is also a key focus of our analysis; we present visualizations of critical steps in both the person identification and intent recognition processes, providing an intuitive demonstration of the effectiveness of the proposed methods. Finally, we conduct ablation studies to validate the effectiveness of the proposed multi-scale bidirectional encoder.

### 5.1. Comparison of Person Identification Results

The results of person identification are reported in Table 3. We report standard multi-class identification metrics (Accuracy, Precision, and Recall) computed on the held-out test runs, i.e., EEG signals that were not used for model training, as described in Section 4.1. We compare our method with representative EEG person identification studies on the same dataset when available, and further evaluate robustness under different channel budgets.

**Figure 4:**
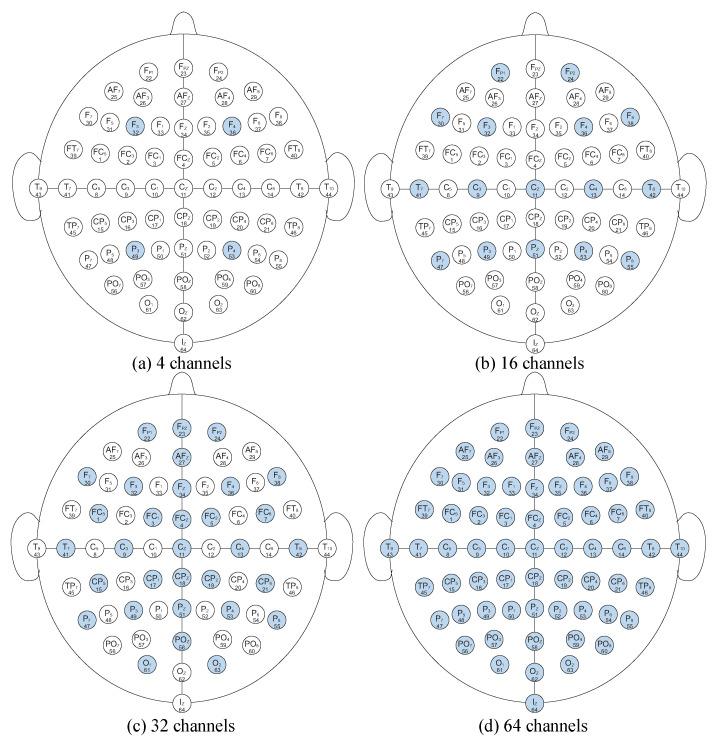
Fixed electrode configurations used by comparison methods under different channel budgets. Blue indicates the selected electrodes (active channels) under each predefined configuration. All baseline models adopt predefined and subject-independent electrode locations, in contrast to the proposed automatic electrode selection strategy.

To assess robustness under different sensing constraints, we conduct experiments using 4, 8, 16, 32, and 64 channels, respectively. For competing methods, the channel locations are fixed and follow the configurations illustrated in Figure 4. In contrast, our method employs the proposed AES module to automatically select a subject-specific set of electrodes, so the selected channel indices may differ across subjects while the channel budget remains the same.

As shown in Table 3, the proposed CNN-Transformer + AES-MBE achieves the best overall identification performance across all channel settings. Notably, under the most constrained setting (4 channels), our method attains an accuracy of 98.82%, outperforming the previous state-of-the-art result (94.71%) by 4.11%. This improvement is most pronounced at low channel counts because AES explicitly prioritizes the most subject-discriminative electrodes for each individual, which reduces the impact of using a small and potentially suboptimal fixed electrode subset. As the number of channels increases (e.g., 32 and 64 channels), performance for most approaches saturates, and the gap narrows due to the increased spatial information and a ceiling effect; nevertheless, our method still achieves the best or tied-best accuracy while maintaining lower error rates.

In addition to identification accuracy, the error metrics (FAR, FRR, and EER) in Table 3 consistently decrease as the channel budget increases, indicating that richer spatial coverage improves discriminability and reduces both false accepts and false rejects. Our method yields low EER across all channel settings (e.g., 1.04% with 4 channels and 0.018% with 64 channels), which is particularly important for security-oriented scenarios. It should be noted that the FAR, FRR, and EER reported in Table 3 are computed based on the verification protocol derived from the multi-class identification outputs, as described in Section 4.1. These metrics are provided to characterize the model’s discriminative capability in a verification scenario, rather than to indicate a separately trained verification system.

**Figure 5:**
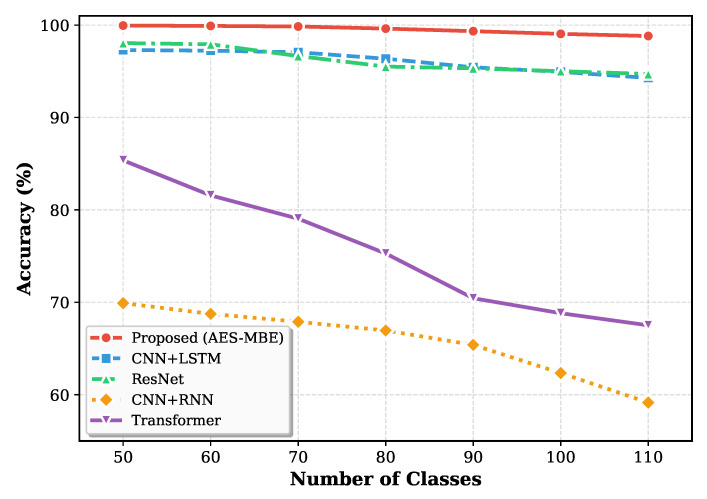
Person identification accuracy as a function of the number of enrolled subjects under the 4-channel setting, used to evaluate scalability and robustness with limited electrodes.

To further examine scalability with respect to the number of enrolled users, Figure 5 shows the identification accuracy as the number of subject classes increases under the challenging 4-channel setting. The proposed method maintains a relatively stable accuracy trend, whereas competing methods exhibit more noticeable degradation, especially when the number of classes becomes large. This suggests that the proposed backbone (AES-MBE) is more effective at extracting subject-discriminative temporal features even under limited spatial sampling, thereby improving robustness and generalization in large-scale identification scenarios.

In addition to comparing different electrode numbers, we further analyze the impact of different electrode selection strategies on person identification performance, including a traditional fixed selection, a common global electrode set shared across subjects, and subject-specific electrode selection. For the common global strategy, we first obtained a subject-specific electrode importance ranking using the AES module for each subject. A unified electrode set was then constructed by counting the frequency with which each electrode appears in the top-*k* ranked electrodes across all subjects, and selecting the top-*N* electrodes (with N=4,8, and 16) with the highest frequencies as the global configuration. The comparison results are presented in Table 4.

As shown in Table 4, both the common global selection and the proposed subject-specific selection outperform the traditional fixed electrode strategy, indicating that data-driven channel selection is beneficial even when a unified configuration is adopted. However, subject-specific electrode selection consistently achieves the best performance across all electrode numbers. Notably, compared with the common global electrode set, the subject-specific strategy yields a performance improvement of 2.19% and 0.77% when using 4 and 8 electrodes, respectively, while the gap becomes marginal when 16 electrodes are used.

This observation suggests that inter-subject variability in EEG spatial characteristics plays a more critical role under low-channel settings, where a unified electrode configuration cannot fully capture individual discriminative patterns. Although a common electrode set may reduce implementation complexity, the proposed subject-specific selection provides clear advantages in resource-constrained scenarios. It is worth emphasizing that electrode selection is performed offline during the enrollment stage and remains fixed during subsequent identification sessions, thereby improving performance without introducing additional online complexity.

### 5.2. Comparison of Intent Recognition Results

Table 5 presents the results of intent recognition in the pre-training stage. In this stage, a separate model is trained for each target subject, and the intent-related labels are used to facilitate learning subject-discriminative attention patterns for electrode selection (Section 4.1.1). Since a separate model was trained for each person during the pre-training phase, the average intent recognition accuracy across all subjects reached 91.58%, with the highest person accuracy reaching up to 99.95%. The high best-subject accuracy suggests that, for some individuals, the proposed backbone can learn a highly separable representation even with the additional “other-subject” negative category, whereas the gap between best-subject and average accuracy reflects inter-subject variability that is well known in EEG-based classification. It is worth emphasizing that, in our framework, this “intent recognition” evaluation is reported only for the pre-training stage and serves as an auxiliary indicator of representation quality to support subject-specific electrode selection, rather than being intended as a protocol-aligned benchmark for conventional intent or motor-imagery recognition. Since intention-related cognitive processes may activate different brain regions depending on the task and the individual, we do not assume that a standardized channel selection strategy is necessarily optimal for intent recognition. Accordingly, the intent recognition task is performed in a subject-dependent manner, and the learned channel attention is used to facilitate subject-specific electrode selection for the downstream person identification task. We also provide a comparison with representative EEG-based intent/motor-imagery recognition studies reported in the literature. However, the compared works may differ in terms of datasets, the number/definition of classes (e.g., five-class motor imagery recognition in many prior studies versus our 4 category pre-training setting that includes non-current subject data), and evaluation protocols (subject-dependent or subject-independent). Therefore, Table 5 is included for contextual reference rather than as a strictly protocol-aligned or directly comparable benchmark. Within this context, the proposed AES-MBE achieves competitive and strong performance under both the 20-subject and 109-subject settings, indicating that the learned representations remain effective when scaling to a larger subject pool.

To further verify the effectiveness and generalization ability of the proposed AES-MBE backbone network, we conducted a series of comparative analyses by replacing our MBE with different baseline architectures and evaluating both intent recognition and person identification performance. This comparison is designed to isolate the contribution of the backbone representation learner, while keeping the downstream training protocol and evaluation principle unchanged. As shown in Table 6, our AES-MBE backbone consistently achieves the highest average accuracy in intent recognition and also demonstrates superior person identification results after electrode auto-selection. Compared with CNN + RNN and a plain Transformer, AES-MBE provides a notable gain in average intent recognition accuracy (e.g., 91.58% versus 84.64% and 83.49% on 109 subjects), which suggests that combining multi-scale temporal encoding with bidirectional modeling yields more robust EEG representations. Importantly, the same ordering is preserved when transferring the learned electrode configuration to the person identification task: stronger intent-related representations lead to more reliable subject-discriminative electrode selection, which in turn improves identification accuracy under limited channel budgets (4 and 8 channels). This optimal performance is attributed to the excellent feature capture capability of the proposed multi-scale bidirectional encoder, which can select more specific and discriminative electrodes for each subject.

**Figure 6:**
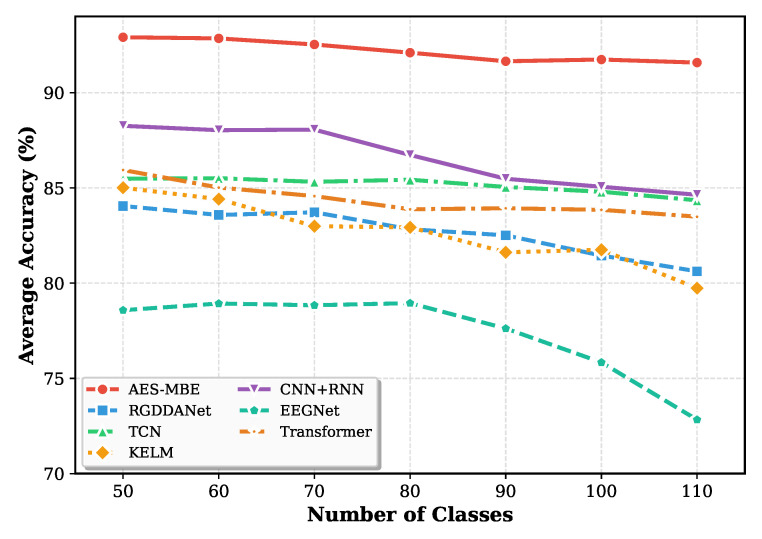
Average intent recognition accuracy versus the number of classes, used to evaluate scalability of different backbone modules.

Furthermore, to validate the scalability of our model, we examined changes in average intent recognition accuracy as the number of classes increased. This setting is intentionally challenging because increasing the number of classes reduces inter-class margins and requires the model to preserve fine-grained temporal cues. The results, illustrated in Figure 6, show that the AES-MBE model maintains stable performance without significant degradation, while other models exhibit noticeable accuracy drops as the number of classes increases. The relatively flat trend of AES-MBE indicates that the proposed backbone better captures both global and local temporal dependencies and mitigates overfitting to a small subset of classes, which is consistent with its multi-scale and bidirectional design. This confirms that the proposed backbone network can more effectively capture both global and local temporal dependencies under large-scale data classification scenarios, thereby achieving superior overall classification performance.

### 5.3. Feature Visualization

In this section, we present qualitative visualizations of neural features extracted by the classification models under different tasks. The goal is to provide an intuitive interpretation of how the proposed AES-MBE backbone progressively increases class separability along the processing pipeline, which complements the quantitative results reported in Table 3, Table 4, Table 5 and Table 6. For person identification, we visualize three stages using 10 selected subjects: (i) the raw EEG feature distribution, (ii) the feature distribution after applying subject-specific automatic electrode selection under a 4-channel budget, and (iii) the final embedding produced by the complete identification pipeline. For intent recognition in the pre-training stage, we visualize one selected subject with 4 categories (“other persons” and three intent-related states for the target subject), and compare the distributions before and after key modules (MBE), illustrating how the model separates the target subject from non-target subjects while preserving intent-discriminative structure.

The overall visualization procedure is as follows:

1. The model is trained according to the described architecture and procedure, with a data subset consisting of 10 selected subjects for person identification visualization and one selected subject for intent recognition visualization.
2. The neural features at each stage of the model pipeline are extracted.
3. t-SNE is used to project the extracted features into a two-dimensional embedding space [52].
4. The features projected into the two-dimensional space are plotted, with each data point color-coded according to its corresponding intent or person category.

We note that t-SNE is employed here to provide an interpretable two-dimensional view of the learned embeddings, and the conclusions drawn from these plots are qualitative rather than statistical.

**Figure 7:**
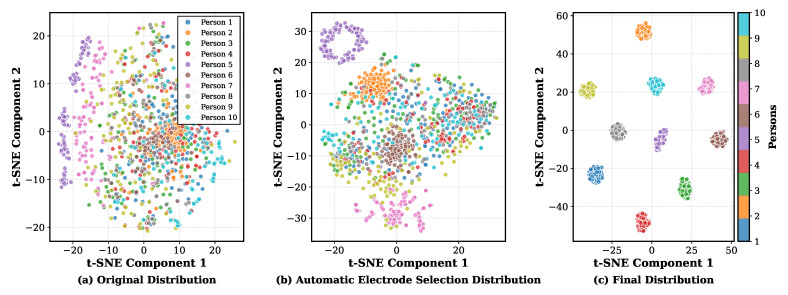
t-SNE visualization of person identification embeddings for 10 selected subjects: (**a**) raw EEG features, (**b**) features after subject-specific automatic electrode selection under a 4-channel budget, and (**c**) final embeddings after the complete identification pipeline.

The feature visualization results for the person identification task are shown in Figure 7. As illustrated in Figure 7a, the raw feature distributions of the 10 selected persons show no clear separability, with extensive overlap among subjects. Some persons, such as Person 5 and Person 7, exhibit large intra-subject variability, which aligns with the inherent characteristics of EEG signals. Moreover, since the proposed system is designed for cross-task person identification, the EEG variability caused by different tasks performed by the same person further increases the difficulty of identification. Figure 7b shows the feature distribution after automatic electrode selection for each person. Compared with Figure 7a, the clusters become more compact and less entangled, indicating that AES reduces task-irrelevant and subject-irrelevant components by focusing on subject-discriminative channels under the 4-electrode constraint. It can be observed that, after applying the AES-MBE framework, a significant amount of irrelevant information is removed, and distinctive features are maximized for each person. At this stage, several persons, such as Person 2, Person 5, Person 6, and Person 7, already exhibit clear clustering with greatly enhanced separability. Figure 7c presents the final feature distribution after passing through the person identification model. It is evident that, after processing by the AES-MBE backbone and the CNN + Transformer model, all persons are distinctly separated into well-defined clusters, which is consistent with the near-ceiling identification accuracy reported in Table 3 even under low channel budgets.

**Figure 8:**
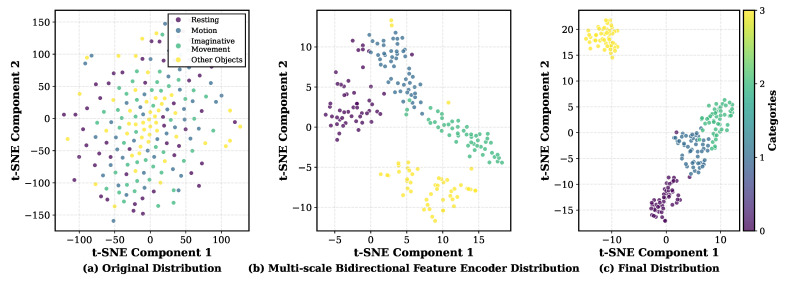
t-SNE visualization of pre-training intent recognition embeddings for one selected subject: (**a**) raw EEG features, (**b**) embeddings after the MBE module, and (**c**) final embeddings after the AES-MBE backbone (4 categories: other persons, motor imagery, motor execution, and resting).

The visualization results for intent recognition are shown in Figure 8. Figure 8a depicts the raw data distribution of a single subject during the pre-training stage, including 4 categories: data from other persons, real motion, imagined motion, and the person’s resting state. The heavy overlap in Figure 8a indicates that intent-related differences are not directly separable in the raw space, motivating the need for a strong temporal representation learner. Figure 8b displays the feature distribution after processing through the MBE. Thanks to the excellent feature extraction and representation capability of the proposed MBE architecture, these 4 categories are already largely separable. In particular, the “other subjects” samples form a distinct region separated from the target subject’s intent-related samples, supporting the role of the pre-training objective in enforcing subject-specific discrimination. We also observe that motor and motor imagery features are closely located, while the resting state features are more distant. Additionally, the features from other persons are positioned the farthest away. This pattern is qualitatively consistent with the intuitive similarity between motor execution and motor imagery, while resting state exhibits a more distinct activity pattern. The same observations apply to Figure 8c, which presents the final output feature distribution from the AES-MBE model. Compared with Figure 8b, AES-MBE further tightens within-class clusters and increases inter-class margins, indicating that combining AES with MBE yields more discriminative embeddings for the pre-training intent recognition objective.

### 5.4. MBE Ablation Experiments

We conduct ablation experiments to quantify the contribution of each component in the proposed multi-scale bidirectional encoder (MBE). As shown in Table 7, “Ablated” denotes the component removed from the full MBE, including the local-scale convolution branch, the global-scale convolution branch, and the forward/backward temporal convolutions. We report performance on both the pre-training intent recognition task and the downstream person identification task (after AES-based electrode selection), to verify whether the same architectural factors consistently benefit the two stages of our framework.

Overall, retaining the complete MBE yields the best results across settings. For intent recognition, removing either the local or global branch leads to a noticeable drop in average accuracy (e.g., from 99.64% to 97.12%/97.50% on 20 subjects, and from 91.58% to 90.01%/90.37% on 109 subjects), indicating that multi-scale temporal modeling contributes complementary information. Similarly, disabling one temporal direction degrades performance (Backward: 97.31%, Forward: 96.84% on 20 subjects; 89.96% and 89.74% on 109 subjects), suggesting that bidirectional encoding provides additional discriminative cues beyond a single-direction convolution.

A consistent pattern is observed for person identification accuracy under limited channel budgets: the full MBE achieves the highest identification accuracy (98.82% with 4 channels and 99.12% with 8 channels), while removing any component reduces performance (e.g., 96.62%–97.19% with 4 channels and 97.56%–98.02% with 8 channels). The relatively similar impact of ablating the forward versus backward branch further indicates that both directions contribute comparably to capturing temporal dependencies in EEG signals.

These ablation results support that each component in MBE plays a distinct role: multi-scale branches enhance robustness by capturing both local and global temporal patterns, while bidirectional convolutions improve separability by leveraging temporal context from both directions, which ultimately benefits electrode selection and the final identification performance.

### 5.5. Limitations and Future Work

Non-causality and real-time deployment. A primary limitation is that the proposed multi-scale bidirectional encoder is not strictly causal, since it leverages future samples within a predefined 1 s window. Therefore, the current design is more suitable for offline analysis or block-based processing than for strict low-latency streaming. In future work, we will investigate (i) causal or forward-only variants of the encoder, (ii) sliding-window implementations with explicit latency control, and (iii) model compression/distillation to obtain a lightweight causal model while maintaining the accuracy of the bidirectional teacher network.

Closed-set assumption and unknown-user handling. All person identification experiments in this study are conducted under a closed-set setting, where test identities are included among the enrolled subjects seen during training. Although we report FAR/FRR/EER via a verification protocol derived from multi-class outputs, the model is still trained as a closed-set classifier and does not explicitly address open-set identification or unknown-user rejection. A practical biometric system must be able to reject unseen users and support incremental enrollment. Future work will extend the framework to open-set scenarios by incorporating explicit unknown-user modeling (e.g., calibrated score thresholding, metric-learning objectives, or prototype-based identification) and by evaluating performance under unseen-subject test splits.

Task dependence of channel selection. Channel relevance can be task-dependent, especially for intent recognition, where distinct brain regions may be involved across different intentions and subjects. In this study, the intent-recognition stage is primarily used to facilitate learning subject-discriminative attention for electrode selection, and we do not claim that the selected electrodes are universally optimal for all intent-recognition paradigms. Future work will explore task-aware selection strategies, including multi-objective electrode selection that jointly optimizes person identification and intent recognition, as well as adaptive selection conditioned on the detected task/activity.

Cross-session and cross-device generalization. EEG biometrics are affected by session variability, electrode placement shifts, and device-specific noise. While the run-wise split mitigates temporal leakage within the dataset, the experiments are still conducted on a single public dataset collected under a consistent acquisition system. Future work will evaluate cross-session and cross-device transfer, and investigate domain generalization/adaptation techniques, including robust normalization, augmentation, and lightweight calibration for new sessions.

## 6. Conclusions

This paper presents a unified deep learning framework for efficient EEG-based biometric interaction, integrating automatic electrode selection, person identification, and downstream intent recognition within a sequential pipeline. To address redundancy and inter-subject variability in multi-channel EEG, we propose the AES-MBE backbone, where the AES module learns subject-specific channel importance and the multi-scale bidirectional encoder captures both local and global temporal dependencies by modeling EEG dynamics in forward and backward directions. Based on the resulting subject-specific electrode configuration, we further introduce a CNN–Transformer person identification model (CMA) that combines local convolutional feature extraction with global dependency modeling via multi-head self-attention.

Extensive experiments on the EEGMMIDB demonstrate that the proposed framework achieves state-of-the-art performance. Notably, under the highly constrained 4-electrode setting, our method attains a person identification accuracy of 98.82% with low verification error rates, highlighting its potential for resource-limited and security-oriented applications. Moreover, the pre-training stage yields strong subject-dependent intent recognition performance, supporting the effectiveness of the learned representations used for electrode selection. Additional analyses show that subject-specific electrode selection consistently outperforms fixed and common global electrode configurations under low-channel budgets, and ablation studies verify that both the multi-scale branches and the bidirectional temporal modeling contribute to the final performance.

Overall, these results suggest that individualized electrode selection combined with robust temporal representation learning can substantially reduce sensing complexity without sacrificing recognition accuracy. This work provides a practical pathway toward portable EEG-based biometric devices and identity-aware EEG interaction systems. Future work will focus on extending the framework to open-set and cross-session scenarios, improving real-time capability via causal modeling, and developing more interpretable and task-aware electrode selection strategies for broader deployment.

## Figures and Tables

**Table 1:** Details of the PhysioNet Dataset.

Item	Description
Number of participants	109
Number of channels	64
Sampling rate	160 Hz
Frequency band	delta: 0.5–3 Hz
	theta: 4–7 Hz
	alpha: 8–13 Hz
	beta: 14–29 Hz
	gamma: 30–47 Hz
Task types	resting state
	moving a single fist
	imagine moving a single fist
	moving both fists or feet
	imagine moving both fists or feet

**Table 2:** Architecture of AES-MBE Backbone.

Layer Type	Layer Characteristics	Output Shape
Input		(256, 64, 160)
Batch normalization	ϵ=0.0001	(256, 64, 160)
Automatic Electrode Selection Module		
GAP		(256, 64, 1)
Squeeze		(256, 64)
Fully connected	64×64	(256, 64)
Sigmoid		(256, 64)
Unsqueeze		(256, 64, 1)
Channel-wise Mul		(256, 64, 160)
Multi-scale Bidirectional Encoder × N		
GAP		(256, 64)
Fully connected	64×64	(256, 64)
Sigmoid		(256, 64)
Unsqueeze		(256, 64, 1)
Forward Local Conv	64×64×3×1	(256, 64, 160)
Sigmoid		(256, 64, 160)
Forward Global Conv	64×64×7×3	(256, 64, 160)
Transpose		(256, 160, 64)
Fully connected	64×64	(256, 160, 64)
Transpose		(256, 64, 160)
Add		(256, 64, 160)
Backward Local Conv	64×64×3×1	(256, 64, 160)
Sigmoid		(256, 64, 160)
Backward Global Conv	64×64×7×3	(256, 64, 160)
Transpose		(256, 160, 64)
Fully connected	64×64	(256, 160, 64)
Transpose		(256, 64, 160)
Add		(256, 64, 160)
Combine		(256, 64, 160)
GAP		(256, 64)
Fully connected	64×16	(256, 16)
Fully connected	16×4	(256, 4)

**Table 3:** Comparison of person identification performance on the PhysioNet dataset under different channel budgets. The proposed method adopts subject-specific automatic electrode selection (AES), while competing methods use fixed electrode configurations. Bold numbers indicate the best performance under each metric.

Method	Dataset	Backbone	Subject	Identification Metrics (%)				Error Metrics (%)		
				Channels	Accuracy	Precision	Recall	FAR	FRR	EER
Benomar et al. [47]	BED [48]	EEGNet	21	14	86.74	89.13	86.68	-	-	-
Jin et al. [32]	THU-EP [49]	MD-CAT	80	32	98.62	98.96	98.62	-	-	-
Buzzelli et al. [11]	EEGMMIDB	CNN + RNN	109	4	59.15	-	-	-	-	-
				64	**99.98**	-	-	-	-	0.39
Sun et al. [10]	EEGMMIDB	CNN + LSTM	109	4	94.28	-	-	5.48	5.71	5.60
				16	99.58	-	-	0.41	0.41	0.41
				32	99.50	-	-	0.49	0.50	0.49
				64	99.58	-	-	0.41	0.42	0.41
Seyfizadeh et al. [12]	EEGMMIDB	ResNet	109	4	94.71	-	-	0.70	7.00	3.85
				16	99.43	-	-	0.095	0.91	0.50
				32	99.51	-	-	0.091	0.83	0.46
				64	99.73	-	-	0.082	0.75	0.41
Proposed	EEGMMIDB	CNN–Transformer + AES-MBE	109	4	**98.82(+4.11)**	**99.06**	**98.53**	**0.68**	**1.17**	**1.04**
				8	**99.12**	**99.30**	**98.75**	**0.18**	**0.87**	**0.43**
				16	**99.62**	**99.41**	**98.92**	**0.09**	**0.38**	**0.25**
				32	**99.74**	**99.80**	**99.49**	**0.09**	**0.26**	**0.16**
				64	**99.98**	**99.98**	**99.12**	**0.018**	**0.019**	**0.018**

**Table 4:** Comparison of different electrode selection strategies for person identification, including fixed selection, a common global electrode set shared across subjects, and subject-specific selection. Bold numbers indicate the best performance under each metric.

Electrodes	Electrode Selection Strategy (%)			Improvement over Common (%)
	Fixed (Traditional)	Common (Global)	Subject-Specific (AES-MBE)	
4	95.02	96.63	**98.82**	+2.19
8	97.85	98.35	**99.12**	+0.77
16	99.58	99.59	**99.62**	+0.03

**Table 5:** Intent recognition results in the pre-training stage (reported as described in the original papers; datasets, class definitions, and evaluation protocols may differ across studies, and the comparison is provided for contextual reference only).

Method	Backbone	Subject	Average Accuracy (%)	Best Subject (%)
Liu et al. [50]	RGDDANet	9	86.25	97.34
Lu et al. [16]	TCN	20	97.89	99.89
Venkatachalam et al. [51]	KELM	5	92.88	96.54
Buzzelli et al. [11]	CNN + RNN	20	98.43	-
		103	85.73	-
Proposed	AES-MBE	20	99.64	99.95
		109	91.58	99.95

**Table 6:** Results of intent recognition (pre-training stage) and person identification (registration stage) using different backbone modules under the same evaluation protocol, highlighting the effect of the proposed AES-MBE. Bold numbers indicate the best performance under each metric.

Module	Subject	Intent Recognition (%)		Person Identification (%)	
		Average Accuracy	Best Subject	Channels	Accuracy
RGDDANet	109	80.62	93.36	4	92.32
				8	94.53
TCN	109	84.35	95.62	4	93.63
				8	95.12
KELM	109	79.74	92.95	4	91.05
				8	92.30
CNN + RNN	109	84.64	98.72	4	94.81
				8	96.57
EEGNet	109	72.83	88.31	4	90.74
				8	94.09
Transformer	109	83.49	95.74	4	95.38
				8	96.75
AES-MBE	109	**91.58**	**99.95**	4	**98.82**
				8	**99.12**

**Table 7:** MBE ablation results on intent recognition (pre-training stage) and person identification (registration stage) after AES-based electrode selection. “Ablated” indicates the removed component from the full MBE; person identification is reported under limited channel budgets (4 and 8 channels). Bold numbers indicate the best performance under each metric.

Ablated	Subject	Intent Recognition (%)	Person Identification (%)	
		Average Accuracy	Channels	Accuracy
Local Conv	20	97.12	4	96.62
	109	90.01	8	97.56
Global Conv	20	97.50	4	96.74
	109	90.37	8	97.81
Backward	20	97.31	4	97.19
	109	89.96	8	98.02
Forward	20	96.84	4	96.85
	109	89.74	8	97.75
-	20	**99.64**	4	**98.82**
	109	**91.58**	8	**99.12**

## Data Availability

The datasets generated and analyzed during the current study are publicly available in the PhysioNet repository: https://physionet.org/content/eegmmidb/1.0.0/ (accessed on 1 January 2026).
